# Sleep disturbance, depression and pain in adults with sickle cell disease

**DOI:** 10.1186/1471-244X-14-207

**Published:** 2014-07-21

**Authors:** Gwenyth R Wallen, Caterina P Minniti, Michael Krumlauf, Ellen Eckes, Darlene Allen, Anna Oguhebe, Cassie Seamon, Deepika S Darbari, Mariana Hildesheim, Li Yang, Jeffrey D Schulden, Gregory J Kato, James G Taylor VI

**Affiliations:** 1 National Institutes of Health, Clinical Center, Bethesda, MD, USA; 2 Genomic Medicine Section, Hematology Branch, National Heart, Lung and Blood Institute, National Institutes of Health, Building 10-CRC, Room 5-5140 MSC 1476, Bethesda 20892-1476 MD, USA; 3 Center for Cancer and Blood Diseases, Children’s National Medical Center, Washington, DC, USA; 4 Division of Epidemiology, Services, and Prevention Research, National Institute of Drug Abuse, National Institutes of Health, Bethesda, MD, USA

**Keywords:** Sickle cell disease, Sleep disturbance, Depression, Chronic pain, Patient reported outcomes, Bethesda sickle cell cohort study

## Abstract

**Background:**

Sleep disturbance and depression are commonly encountered in primary care. In sickle cell disease, depression is associated with pain, poor treatment compliance, and lower quality of life. The prevalence of sleep disturbance and its effect upon quality of life in adults with sickle cell disease is unknown. The goal of this study was to determine the prevalence of sleep disturbance and if it is associated with pain and depression in sickle cell disease.

**Methods:**

Three hundred twenty eight adults with sickle cell disease enrolled on the Bethesda Sickle Cell Cohort Study were assessed using the Pittsburgh Sleep Quality Index and Beck Depression Inventory II screening measures as a cross-sectional survey. Scores greater than 5 (Pittsburgh Sleep Quality Index) and 16 (Beck Depression Inventory II) defined sleep disturbance and depression, respectively. Clinical and laboratory parameters were also assessed.

**Results:**

The mean Pittsburgh Sleep Quality Index score was 8.4 (SD ± 4.2) indicating a 71.2% prevalence of sleep disturbance. The mean Beck Depression Inventory II score was 8.0 (SD ± 8.9). Sixty five (20.6%) participants had a score indicating depression, and half of these (10.0%) had thoughts of suicide. Both Pittsburgh Sleep Quality Index and Beck Depression Inventory II scores were significantly correlated (p < .001). The number of days with mild/moderate pain (p = .001) and a history of headaches (p = .005) were independently associated with depression by multivariate regression analysis. Patients with sleep disturbance were older (p = .002), had higher body mass index (p = .011), had more days of pain (p = .003) and more frequent severe acute painful events (emergency room visits and hospitalizations) during the previous 12 months (p < .001).

**Conclusions:**

More than 70 percent of adults with sickle cell disease had sleep disturbance, while 21 percent showed evidence of clinical depression. Sleep disturbance and depression were correlated, and were most common among those with more frequent pain. Providers caring for adults with sickle cell disease and frequent pain should consider screening for these common co-morbidities. Additional study is needed to confirm these findings and to determine if treatments for pain, depression or sleep disturbances will improve quality of life measures in this patient population.

**Trial registration:**

ClinicalTrials.gov identifier: NCT00011648.

## Background

Sickle cell disease (SCD) is a recessive genetic disorder caused by mutations in the β-hemoglobin gene on chromosome 11. It affects an estimated 100,000 people in the United States, making it among the most common rare diseases in this country [1,2].

The pathophysiological basis for the clinical manifestations of SCD is polymerization of deoxygenated sickle hemoglobin within the red blood cell, which promotes red cell rigidity and initiates a cascade of events obstructing blood flow in post-capillary venules known as vaso-occlusion. Sickle cell vaso-occlusion can produce intense acute pain, presumably due to tissue hypoxia secondary to obstructed blood flow [3]. Acute, severe painful episodes often necessitate hospitalization for pain management with intravenous opioids. Variability is observed among SCD patients for both the frequency and severity of these painful episodes [4]. In addition to pain, SCD is characterized by chronic organ damage, hemolytic anemia and premature mortality [3].

Depressive disorders, sleep disturbance and fatigue are proposed to be key variables in a biobehavioral model of SCD [5]. Cognitive behavioral processes such as depression and catastrophizing contribute to variation in pain perception and are hypothesized to play a role in pain modulation [6]. The prevalence of depression in SCD ranges from 18 to 44% in adults [7-10], which is substantially higher than an overall prevalence of 9% across the United States [11]. Depression in SCD is associated with greater daily pain, lower quality of life measures, and poor adherence to treatment regimens. Sleep disturbance may be associated with serious, high morbidity SCD complications [12-14], although it is also a well described risk factor for major depression in otherwise healthy individuals [15]. Despite a higher prevalence of high morbidity sleep disturbances like obstructive sleep apnea in SCD, this work has largely focused on children with SCD [12-14,16,17]. As with depression, vaso-occlusive pain crises further exacerbate disturbed sleep and daytime functioning in SCD [18]. In addition, high somatic symptom burden (SSB) in SCD is associated with more non-crisis pain and healthcare utilization by patients seeking symptomatic pain relief. High SSB is also associated with depression, anxiety and poorer health related quality of life [19]. Importantly, the prevalence of sleep problems and their relationship to depression have not been defined in adults with SCD.

The objective of this study was to determine the prevalence of sleep disturbance and the nature of the relationships between depression, sleep disturbance and associated factors defined by patient reported outcomes in a cross-sectional sample of adults with SCD. We used a conceptual explanatory model of pain and healthcare utilization to determine if a relationship exists between disease-related variables, markers of SCD pain, sleep disturbance and depression. Smith and colleagues developed this model of pain and utilization in SCD to examine the relationship of individual demographics, disease-related variables, and psychosocial variables, and their effects on distress disability, healthcare utilization, and pain [20]. For a variety of social and financial reasons, the care of adults with SCD is often provided by primary care physicians [21] who may be ideally suited for screening and treating quality of life co-morbidities that may be overlooked by subspecialists treating these complex patients.

## Methods

### Participants

Three hundred twenty eight subjects undergoing evaluation on a protocol for evaluation of SCD (the Bethesda Sickle Cell Cohort Study; ClinicalTrials.gov identifier NCT00011648) were asked to complete survey instruments as part of a cross sectional sub-study to assess mood and sleep quality during an outpatient evaluation at the NIH Clinical Center between June, 2009 and July, 2012. Survey participants were either 1) undergoing an initial evaluation at NIH or 2) returning for a follow-up evaluation where subjects repeat the same clinical evaluation at regular 2 year intervals from the date of initial protocol enrollment. Those participating in the survey during 2 year follow-up evaluations were originally enrolled on the Bethesda Sickle Cell Cohort Study between March, 2001 and May, 2008 (a total of 488 subjects were enrolled at that time). Subjects were evaluated in steady state without an acute event in the 2 weeks prior to evaluation. This protocol was approved by the National Heart, Lung and Blood Institute Institutional Review Board. All subjects provided written informed consent according to the Declaration of Helsinki.

This sub-study was powered to detect associations with depression where a given factor would double the risk for depression. Assuming a 20% prevalence of depression in SCD [7], a survey of 300 subjects would give 80% power to detect associations of this magnitude at a 1 sided type I (α) error rate of 0.05. Power estimates were not made for sleep disturbance, as its prevalence was unknown in this disease population.

### Clinical history and measures

Subjects completed a medical history survey of patient reported outcomes, with specific inquires about common SCD complications including episodes of acute chest syndrome (ACS) and stroke. Current medications were obtained by patient history. For this study, current narcotic use was defined as either daily sustained release narcotic pain medication use or as needed narcotic pain medication use for acute pain relief during the past month. Clinical evaluation included a physical exam, pulse oximetry and standard laboratory testing. The diagnosis of SCD was established by either DNA sequencing or high performance liquid chromatography/clinical history and was further sub-typed as homozygous SS, combination of Hb S with β-thalassemia-zero (HbSβ^0^; generally severe) or compound heterozygous SCD (generally clinically milder; including HbS with HbC (HbSC) or β-thalassemia-plus (Sβ^+^) [22,23].

### Self-reported pain survey, mood and sleep quality measures

Patients completed a self-reported survey of the number of painful events attributed to SCD during the 12 months prior to evaluation. This survey included 4 pre-defined categories of painful episodes: mild, moderate, severe and extremely severe. Pain episodes were classified as “mild” when the participant reported experiencing pain that did not prevent normal activities and may or may not have required pain medication. A “moderate” episode required pain medication and disrupted normal daily activities (e.g. missed work). Episodes which required an emergency department visit without hospitalization were classified as “severe”. “Extremely severe” acute painful events were the painful episodes which lead to inpatient hospitalizations.

The Beck Depression Inventory (BDI-II) screens for the presence and severity of depression in adults [24]. Each of the 21 BDI-II items queries a particular aspect of depression. Individual items are rated on a 4 point intensity scale rather than on a frequency dimension. A rating of 3 indicates most severe intensity, while 0 indicates the absence of a problem. Thus, the greater the score, the more depressed the individual [25]. BDI-II has been validated in elderly adults with internal consistency (α = 0.91) and predictive validity reported to be high (0.85) [26]. Cut-off scores for depression vary by study with scores between 14 and 17 indicating mild depression and scores ≥20 indicating severe depression [7,8]. In SCD, Asnani and colleagues chose a BDI-II cut-off score of 17 rather than 14 because they found that the higher score would produce greater specificity [7]. A BDI-II cut-off score of ≥17 was also used for this study to define depression. Subjects reporting suicidal thoughts on the BDI survey were evaluated by a psychiatrist or social worker during the clinic visit.

The Pittsburgh Sleep Quality Index (PSQI) is a self-rated questionnaire which provides a measure of sleep quality and disturbances over a month time interval. Nineteen survey items generate seven “component” scores: subjective sleep quality, sleep latency, sleep duration, habitual sleep efficiency, sleep disturbances, use of sleep medications, and daytime dysfunction. Buysse et al. reported a global PSQI score > 5 had a sensitivity of 89.6% and specificity of 86.5% (kappa = 0.75, *p* < .001) in distinguishing subjects with good sleep quality from those with poor sleep [27].

### Statistical analysis

Associations with sleep quality or depression were first examined by dichotomizing subjects into either a depression group (BDI-II score ≥ 17) or a sleep disturbance group (PSQI score ≥ 6) at thresholds previously shown to be predictive of depression and poor sleep, respectively [7,27]. Demographic, clinical, and laboratory characteristics were compared between depressed subjects and subjects without depression using the non-parametric Wilcoxon rank-sum test for continuous variables and Pearson chi-square for categorical variables. Comparisons were made between low and high sleep disturbance groups. Characteristics for analysis were chosen a-priori based on hypothesized associations with pain and sleep in SCD including age, body mass index, frequent headaches, acute chest syndrome and acute painful episodes. Analysis of variance was used to examine associations of BDI-II and PSQI scores with the number of self-reported pain episodes categorized into three groups. Then analysis of covariance was used to examine these associations in multivariate models while adjusting for other factors. For all analyses, covariates were log-transformed as necessary to reduce the influence of outlying values and to meet normality assumptions of the statistical models. Statistical models using different combinations of covariates were generated for multivariable analyses, although only statistically significant variables were presented in the final models. P-values ≤ .050 were considered significant. All analyses were performed using SAS version 9.1.3 (SAS Institute Inc, Cary, NC), Stata version 9.0 (StatCorp LP, College Station, TX) and SVS (Golden Helix, Bozeman, MT).

## Results

**Figure 1:**
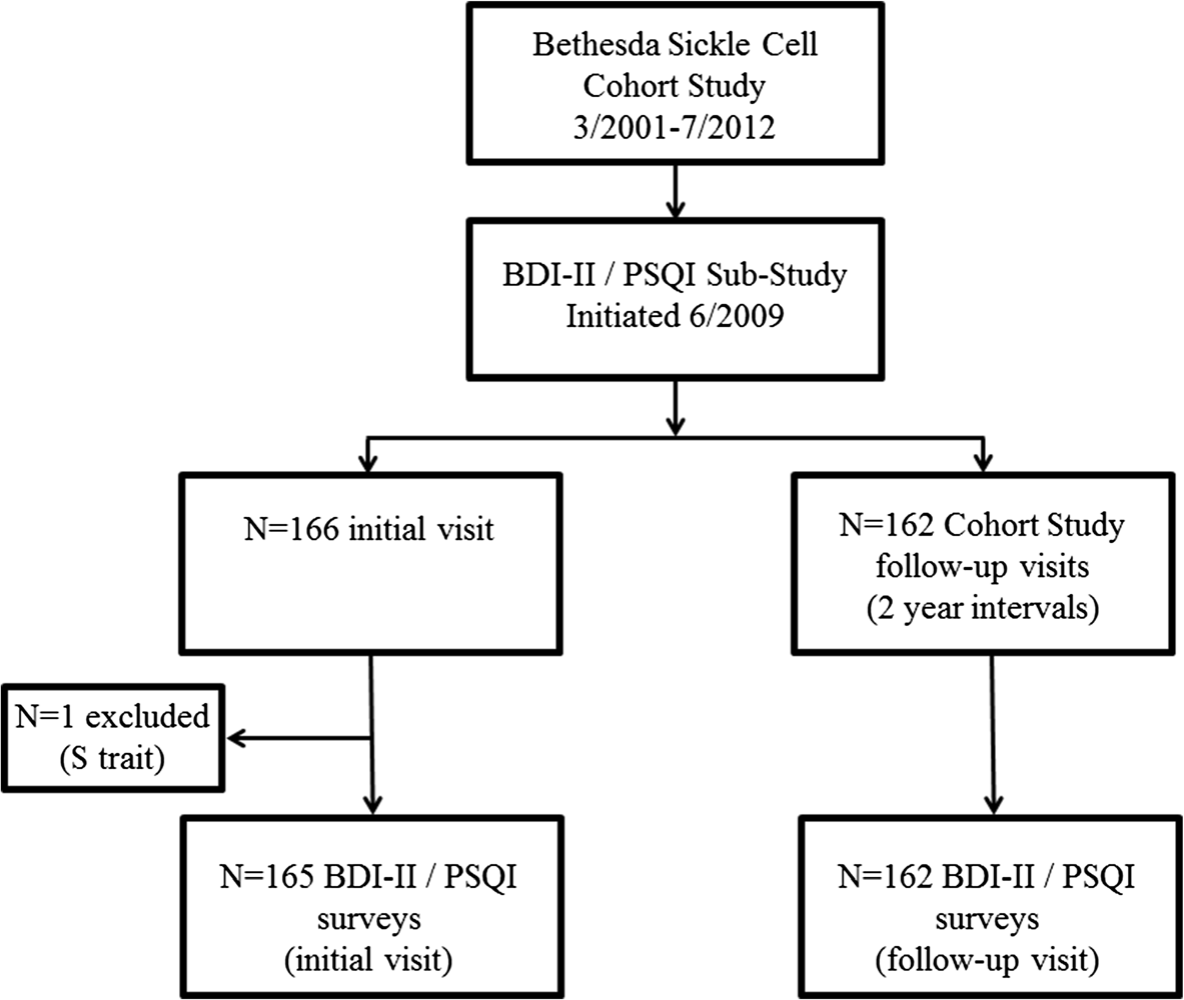
Summary of the Pittsburgh Sleep Quality Index (PSQI)/Beck Depression Inventory II (BDI-II) cross-sectional surveys administered during visits for the Bethesda Sickle Cell Cohort Study.

A total of 328 subjects met criteria for inclusion from 329 surveyed. These 328 subjects represented 50% of the 654 potentially eligible participants enrolled in the Bethesda Sickle Cell Cohort Study during the period of the survey. One subject was excluded from analysis after screening because of a diagnosis of sickle cell trait. One hundred sixty six (51%) completed the surveys at study entry and 162 (49%) did so at a protocol follow-up visit (median time on study 4.7 years, interquartile range (IQR) 2.3 - 7.8) (Figure 1). Follow-up visits were from a pool of 488 subjects enrolled on the Cohort study at the time the survey sub-study was initiated, with 33% of the original cohort completing surveys (162/488). Overall, the population had an even sex distribution (51% female), 277 (85%) with a diagnosis of SS or Sβ^0^ thalassemia and 155 (47%) reported taking hydroxyurea. The median age at the time of surveys was 34 years (IQR 27 – 46), although 67 subjects (20%) were 50 years of age or older. Subjects reported a median of 13.0 (IQR 2.0 -80.0) mild to moderate acute pain days and 2.0 (IQR 0.0 – 6.0) severe to extremely severe pain events over the 12 months prior to evaluation.

**Figure 2:**
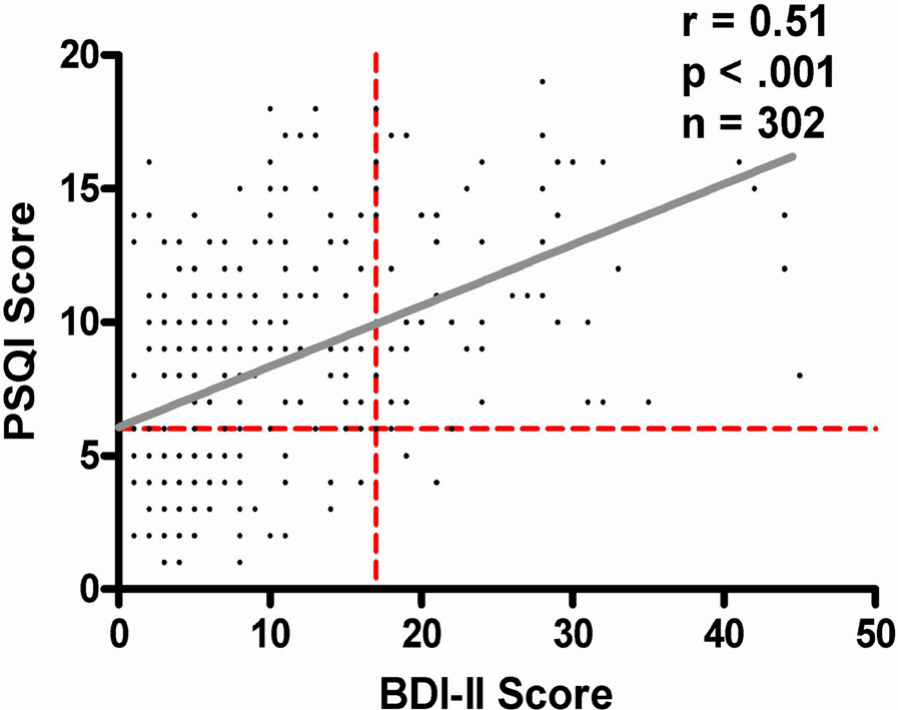
Correlation between Pittsburgh Sleep Quality Index (PSQI) and Beck Depression Inventory II (BDI-II) survey scores.

Depression and sleep disturbance were present in our survey population (Table 1). Three hundred fifteen subjects (96% of sample population) completed a BDI-II survey with a median score of 8 (IQR 4–15). Sixty five (21%) had scores suggestive of depression, including 32 (10%) with suicidal thoughts (BDI-II item 9) (Table 1). Three hundred thirteen PSQI surveys were completed (95% of sample population) with a median score of 9 (IQR 5–11). Two hundred twenty three subjects (71%) had some degree of sleep disturbance (Table 1). All subjects with sleep disturbance reported difficulty sleeping due to pain (PSQI item 5i).For those completing both surveys (n = 302), BDI-II and PSQI scores were significantly correlated (Spearman r = 0.51, p < .001) (Figure 2). Comparison of median scores by Wilcoxon rank-sum gave a comparable result (p < .001, data not shown), where 97 percent (n = 60 of 62) reporting symptoms of depression also had poor sleep.

**Table 1:** BDI-II Depression and PSQI Sleep Scores in Adults with Sickle Cell Disease

**BDI-II**	**SCD Population (n = 328)**
Surveys completed, n (%)	315 (96)
Median (IQR)	8 (4–15)
Score ≥ 17, n (%)	65 (21)
Suicidal thoughts, n (%)	32 (10)
PSQI	
Surveys completed, n (%)	313 (95)
Median (IQR)	9 (5–11)
Score ≥ 6, n (%)	223 (71)
Trouble sleeping due to pain, n (%)	235 (75)

The clinical characteristics of subjects grouped into BDI-II depression and PSQI sleep disturbance groups were then compared to subjects without depression or sleep disturbance, respectively (Table 2). The BDI-II depression group (n = 65) had a significantly higher prevalence of headaches (p = .007), more days of mild/moderate pain (p = .040), more severe/extremely severe pain events (p = .009) and lower hemoglobin (p = .050) than the non-depressed group (Table 2). Similar associations were observed for the sleep disturbance group with PSQI scores ≥ 6 (n = 223) with more prevalent headaches (p < .001), mild/moderate pain (p < .001) and severe/extremely severe painful events (p < .001) (Table 2). In addition, those in the sleep disturbance group were significantly older (p = .009) and had higher body mass index (BMI, 24 versus 23, p = .016). There were no significant differences in hydroxyurea treatment, narcotic use or percentage fetal hemoglobin when comparing either the depression group or the sleep disturbance group to their respective reference groups (Table 2).

**Table 2:** Clinical Characteristic Comparisons by BDI-II Depression and PSQI Sleep Disturbance Groups

**Demographic Variables**	**BDI < 17**		**BDI ≥ 17**		***p***^***1***^	**PSQI < 6**		**PSQI ≥ 6**		***p***^***1***^
	**N**	**Median (IQR) **^**2**^	**N**	**Median (IQR) **^**2**^		**N**	**Median (IQR) **^**2**^	**N**	**Median (IQR) **^**2**^	
Age	250	34 (27–47)	65	35 (27–45)	.95	90	31 (23–45)	223	36 (29–47)	.009
BMI (kg/m^2^)	245	24 (22–27)	63	24 (21–27)	.12	89	23 (20–27)	217	24 (22–28)	.016
Female, N (%)	250	119 (48)	65	40 (62)	.052	90	42 (47)	223	118 (53)	.32
SS/Sβ^0^ thalassemia, N (%)	250	212 (85)	65	53 (82)	.57	90	78 (87)	223	184 (83)	.40
**Clinical History**										
Headache, N (%)	250	92 (37)	64	36 (53)	.007	90	24 (27)	222	105 (47)	<.001
Stroke, N (%)	230	32 (14)	56	6 (11)	.66	81	14 (17)	204	23 (11)	.18
Acute Chest Syndrome, N (%)	248	201 (81)	65	52 (80)	.86	90	70 (78)	223	184 (83)	.34
Hydroxyurea therapy, N (%)	250	120 (48)	65	31 (48)	>.99	90	42 (47)	223	106 (48)	.90
Mild + Moderate Pain (days)	244	11 (2–72)	60	25 (4–126)	.040	88	4 (2–24)	214	23 (4–120)	<.001
Severe + Extreme Pain (events)	250	2 (0–5)	65	3 (0–10)	.009	90	0 (0–2)	223	3 (0–9)	<.001
Current Narcotic Use, N (%)	250	159 (64)	65	47 (72)	.24	90	53 (59)	223	153 (69)	.11
**Laboratory Measures**										
Hemoglobin (g/dL)	250	9.1 (7.8-10.4)	65	8.5 (7.4-9.8)	.050	90	9.3 (8.1-10.4)	223	8.9 (7.6-10.3)	.20
Hemoglobin F (%)	250	5.7 (1.9-12.5)	65	6.0 (3.1-10.7)	.97	90	5.1 (1.8-13.2)	223	6.0 (2.7-11.7)	.76
Oxygen Saturation (%)	248	97 (95–99)	65	97 (95–99)	.97	89	98 (96–99)	222	97 (95–99)	.093

^1^Pearson’s chi-square or Fisher’s exact test for categorical comparisons; Wilcoxon rank-sum for comparison of medians.

^2^Unless otherwise indicated.

The association between sleep disturbance and acute SCD painful events appeared plausible based upon the uniform response to PSQI item 5i (difficulty sleeping due to pain, Table 1) and a higher median number of severe or extremely severe acute painful events in those with PSQI scores ≥6 (p < .001, Table 2). Further scrutiny of this association showed that pain groups defined by the number of severe and extremely severe acute painful episodes had higher mean PSQI scores with increasing numbers of acute painful events (p < .001, Table 3). Increasing mean scores using the BDI-II were also associated with more frequent severe/extremely severe acute painful events (p < .001, Table 3). Analysis of covariance including variables that were significantly associated with BDI-II scores in Table 2, revealed only days of mild/moderate acute pain and a history of headaches were independently associated with higher BDI-II scores (Table 4). A second multivariable analysis of significant variables associated with the sleep disturbance group in Table 2 showed acute painful events (both days of mild + moderate pain and severe + extremely severe pain events), increasing age and BMI were independently associated with higher PSQI scores (Table 5).

**Table 3:** Higher BDI-II and PSQI Survey Scores Associated with More Frequent Severe SCD Painful Events

**Severe + extremely severe pain events Last 12 months**	**BDI-II Score**	**PSQI Score**
	**Mean (95% CI)**	**Mean (95% CI)**
0	8.8 (7.4-10.3)	7.6 (6.8-8.3)
1-4	10.4 (8.5-12.3)	8.2 (7.4-9.0)
5 or more	13.1 (11.2-14.9)	10.3 (9.5-11.0)
*p*^*1*^	<.001	<.001

^1^From ANOVA F-test for comparison of more than 2 groups.

**Table 4:** Independent variables associated with positive screening for depression in SCD

**Variable**	**F (df)**	**p**
Mild + Moderate Pain (days)^1^	10.93 (1,301)	.001
History of Headaches	8. 17 (1,301)	.005

^1^Transformed using the natural log function.

**Table 5:** Independent variables associated with positive screening for sleep disturbance in SCD

**Variable**	**F (df)**	**p**
Mild + Moderate Pain (days)^1^	9.09 (1,290)	.003
Severe + Extreme Pain (events) ^1^	15.62 (1,290)	<.001
Age (years)	9.39 (1,290)	.002
BMI (kg/m^2^)^1^	6.55 (1,290)	.011

^1^Transformed using the natural log function.

## Discussion

This is the first study documenting a high prevalence of sleep disturbance in a cross sectional sample of unselected adults with SCD and the relationship between disordered sleep and depression. These findings suggest that while physical symptoms of SCD, including pain and end organ damage, are the primary clinical focus of providers in outpatient clinics and emergency departments, disrupted sleep architecture and depression are also co-morbid diagnoses in SCD.

Depression and sleep disturbances have been associated with adverse disease trajectories in children and adults [10,28]. Although both of these studies assessed depression using the Patient Health Questionnaire and the SF-36 rather than a depression screening instrument, our findings in adult SCD patients attending an ambulatory clinic are similar, in that approximately one quarter (27.6%) of their sample were ‘depressed’ while 13.8% had major depression. We observed subjects with depression had significantly more self-reported severe pain events (i.e. emergency room visits and hospitalizations). Interestingly, the Pain in Sickle Cell Epidemiology Study (PiSCES) observed that patients with depression had pain on significantly more days than non-depressed participants [10]. Our findings in adults with SCD support the premise from Dampier and colleagues in the Comprehensive Sickle Cell Centers (CSCC) Clinical Trial Consortium (CTC) study of health-related quality of life (HRQOL), namely that improved treatments for pain and depression may provide the largest HRQOL benefit [29]. Furthermore, it remains unclear if improved SCD pain management alone will have a cause and effect relationship to lower the prevalence of depression.

Importantly, we also found that poor sleep is very common and associated with both depression and more frequent pain. There was a significant relationship between subjects reporting PSQI defined poor sleep and those reporting five or more episodes of severe or extremely severe pain episodes in the previous year. BMI was also independently associated with sleep disturbance in this cohort. Previous studies have described the relationship between sleep fragmentation, impaired energy metabolism and BMI [30,31]. The relationship between obstructive sleep apnea and BMI has been well documented [31], yet this relationship has not been studied in SCD. Obesity was not an overall concern in this study with a median BMI of 24 in sleep disturbed subjects, however the significant association between BMI and sleep disturbance may provide areas for further categorization and exploration of associations in future studies. Whether the association between BMI and sleep disturbance in our SCD cohort promotes sleep disturbance or is a consequence of disordered sleep will require further investigation.

As expected, nearly all subjects in our sample who were depressed also reported poor sleep (97%), consistent with sleep disturbance as a symptom of depression. It is interesting to note that, despite the fact that 64.6% of our sample reported chronic narcotic use, self-reported opioid use was not significantly correlated with depression or poor sleep. These findings support previous studies in chronic pain patients without SCD which suggested poor sleep may lead to a more painful day [32,33]. Overall, these data suggest that SCD patients with sleep problems should be screened for depression.

Despite using a set of classical biomarkers associated with SCD severity, only anemia was associated with higher risk for depression (Table 2). Hemoglobin, hematocrit and fetal hemoglobin levels are traditional markers associated with modulating the frequency of SCD pain crises [4]. However, the association between depression and anemia just met criteria for significance, suggesting that the magnitude of this effect is relatively small compared to the association of higher hematocrit with vaso-occlusive pain [4]. None of these parameters were associated with sleep disturbances. Overall, we advocate for universal depression and sleep disturbance screening in adults with SCD, irrespective of genotype (i.e. SS vs. SC) or disease severity.

Neurobiological sleep signaling could potentially be disrupted by frequent antidepressant and narcotic treatment in SCD. Selective serotonin reuptake inhibitors (SSRIs) increase extracellular levels of serotonin while tricyclic antidepressants increase the extracellular levels of serotonin and norepinephrine; both decrease REM sleep [34]. The effect of opioids on sleep architecture is also important to consider since management of pain crisis and chronic pain in sickle cell patients requires variable regimens of narcotics for pain management. Studies have shown that even single doses of oral opioid medications can affect sleep architecture in healthy adults by substantially decreasing their time in deep sleep, stages 3 and 4; while increasing periods of light sleep (stage 2) [35]. Because 65% of the subjects in our study were taking chronic narcotics at the time of the survey, this may contribute to the high prevalence of sleep disturbance in SCD.

These data should be interpreted with several caveats. First, data collected in these survey instruments were patient reported outcomes and not objective measures of depression and sleep quality. If discrepancies exist between self-reported and objective measures, these differences will need to be adjusted so that the associations between sleep and other psychosocial factors are not overestimated [36]. Furthermore, our cross-sectional analysis did not include a measure of somatization which may be a confounding variable in assessing the prevalence of depression in this population. Studies now suggest that somatization, an overwhelming attention to bodily symptoms, in SCD patients is predictive of negative psychological outcomes including depression, anxiety and hostility [19,37].

## Conclusion

Overall, 71% of adults with SCD surveyed reported sleep disturbance, and one in five were depressed. Sleep disturbance and depression assessed by surveys show a moderate correlation (r = .51) and were more common among those with more frequent painful episodes. These findings need to be confirmed by more direct measurements of sleep architecture. Additional study is needed to determine if treatment of pain, depression or sleep disturbance can improve outcomes or health related quality of life in adults with SCD. Providers caring for adults with SCD and frequent pain should consider screening for these common co-morbid conditions.

## Abbreviations

BDI-II: Beck depression inventory II; BMI: Body mass index; IRB: Institutional Review Board; NIH: National Institutes of Health; PSQI: Pittsburgh sleep quality index; SCD: Sickle cell disease; SSB: Somatic symptom burden.

## Competing interests

The authors declare that they have no competing interests.

## Pre-publication history

The pre-publication history for this paper can be accessed here:

http://www.biomedcentral.com/1471-244X/14/207/prepub
